# Stress Impact of COVID-19 Sports Restrictions on Disabled Athletes

**DOI:** 10.3390/ijerph182212040

**Published:** 2021-11-16

**Authors:** Giovanni Fiorilli, Andrea Buonsenso, Nicola Davola, Giulia Di Martino, Francesca Baralla, Stefanos Boutious, Marco Centorbi, Giuseppe Calcagno, Alessandra di Cagno

**Affiliations:** 1 Department of Medicine and Health Sciences, University of Molise, v. De Sanctis 1, 86100 Campobasso, Italy; fiorilli@unimol.it (G.F.); andreabuonsenso@gmail.com (A.B.); francesca.baralla@unimol.it (F.B.); giuseppe.calcagno@unimol.it (G.C.); 2 Department of Economics and Management, LUISS Guido Carli, Viale Romania 32, 00197 Rome, Italy; nicoladavola93@gmail.com; 3 Department of Motor, Human and Health Sciences, University of Rome “Foro Italico”, Lauro de Bosis Square, 15, 00197 Rome, Italy; giulia.dimartino21@gmail.com (G.D.M.); alessandra.dicagno@uniroma4.it (A.d.C.); 4 Department of Physical Education and Sport Science, National and Kapodistrian University of Athens, 15772 Athens, Greece; stefanosboutios@gmail.com

**Keywords:** adapted sport, COVID-19 pandemic, psychological distress, well-being

## Abstract

The stress impact of COVID-19 restrictions has put the adapted sports community at an unprecedented level of emergency. The self-report Event Scale—Revised (IES-R) questionnaire was administered to assess the level of psychological distress and emotive reactions such as intrusion (INT), avoidance (AV) and hyperarousal (HYP) following training and competitions suspension within a sample of Italian disabled athletes. A total of 146 self-selected volunteers were included in this study: 73 disabled athletes (aged 42.11 ± 13.70) and 73 athletes (aged 40.23 ± 13.73) who served as the control group. Only 8.22% of the disabled participants vs. 30.14% of athletes were affected by subjective distress. MANOVA showed significant differences in IES-R subjective distress for age, where the emerging adults had a higher level of stress than adults (*p* = 0.031), and for the type of sport, where the individual sports group showed higher scores than the team sports group (*p* = 0.049). Regarding the IES-R subscales, significant differences were found in INT and AV for age, where the emerging adults showed higher scores than adults (*p* = 0.018 and *p* = 0.046, respectively). Significant differences were found in HYP for type of sport, where the individual sports group showed higher scores than the team sports group (*p* = 0.014). The results confirmed a lower distress level of disabled athletes to adverse events than that expressed by athletes. Both sports engagement and the experience of living with impairment, overcoming structural barriers, could act as a buffer effect against stress due to COVID-19 restrictions.

## 1. Introduction

The physical, mental and social well-being benefits of sports and physical activity for disabled people are well recognized, such as maintaining a healthy weight, improving fitness and physical self-efficacy and enhancing body image and independence, as well as developing friendships [1]. “Adapted” sports activity, originating in the relatively recent period, provides valuable assistance to promote independent living and the social inclusion of people with disabilities [2,3,4]. Athletic endeavors in such populations ensure greater mental and motor benefits than people without disabilities [5]. Disabled athletes recognize their physical and mental self-empowerment and perceived well-being due to sports training participation, gaining a “strong athletic persona” [6].

Recognizing these benefits, state agencies in several countries have placed emphasis on the increased participation of disabled people in sport, and this program has become an important issue in health policy [7]. In Italy, it is believed that Paralympic sports involve approximately 14,650 athletes, 1824 coaches and 3700 managers [8].

In March 2020, the global SARS-COVID-19 pandemic led to several restrictions in Italy, enforcing policies regarding the avoidance of human-to-human transmission, via social distancing [9]. These restrictions have changed the daily routine of people forced to stay at home, limiting interpersonal interactions and any type of physical activity [10]. The closure of recreational and sportive centers, such as schools and gyms, led to changes in athletes’ daily training and scheduled competitions, influencing their physical, technical and psychological development [11].

In particular, COVID-19 restrictions have affected disabled athletes, who have experienced a lack of opportunities and programs for training and competitions and accessible information. Performing physical activity is mandatory for people with disabilities who frequently present with secondary diseases such as coronary heart disease, diabetes and obesity and allows preventing COVID-19 complications [12]. Specifically, their disability conditions may place them at a greater risk of complications from COVID-19 [9], which can increase perceived stress. Overall, people with disabilities commonly experience social participation disparities compared to people without disabilities. For this reason, they are a socially vulnerable (not implicitly vulnerable) and marginalized group [13], whose social participation may be disproportionately affected by lockdown-related measures [14]. Additionally, the abrupt interruption of disabled athletes’ daily routines, life-altering events and stay-at-home orders related to the COVID-19 pandemic may overwhelm their individual ability to cope and increase anxiety, fear and negative emotions [15,16]. Fitzgerald and collaborators [17] explored how sport is important within the context of COVID-19 restrictions, but few observations were of interest to disabled athletes.

Therefore, the impact of COVID-19 restrictions disproportionately puts the disabled sports community at risk of distress, and long-lasting effects may be expected [18].

Specifically, from the lifespan perspective, their existing disabilities and poorer health status may increase perceived stress and exacerbate health and well-being among individuals with disabilities and chronic conditions [19].

Moreover, the abrupt interruption of their daily routines, social contacts and the possibility to overcome their limits and barriers through sport could be perceived by disabled athletes as a traumatic event that overwhelms their individual ability to cope. Psychological trauma could be defined as the individual experience of an event or enduring conditions of this event that overwhelms the individual’s ability to integrate their emotional experience. “It is an event perceived by the subject as “critics”, generating impotence and vulnerability, capable of causing such severe stress to threaten the psychophysical balance” [20].

Several researchers have underlined that perceived stress is related to poorer health status and a higher level of distress, anxiety and depression in individuals with disabilities [21]. It has been shown that physical impairments could be risk factors for post-traumatic stress disorder development. It was found in [22,23] that 44% of people with paraplegia showed a diagnosis of lifetime post-traumatic stress disorder.

Despite the challenges related to the disabilities, many disabled people have reported, over time, the development of a positive perspective in their lives (growth-oriented perspective), even related to supportive physical, social and emotional environments [24]. As Hockenberry [25] underlined: “From the beginning, disability taught that life could be reinvented”. In this direction, empirical studies have shown that disabled people experience living with impairment and, frequently, affirm that the presence of disability made them stronger, being forced to face “at-risk” situations [26] and challenging social and structural barriers [27]. Moreover, disabled people also learn to take advantage of potential external support, provided by society or their families, to overcome their own vulnerabilities [28,29]. Therefore, many people with disabilities report having stable moods, even after experiencing disability-related stresses [30]. In fact, a sort of developed resiliency gives them the inner strength to face and deal with adversities and risks [31].

A self-report questionnaire for the evaluation of perceived stress, the Impact of Event Scale—Revised (IES-R) from Weiss and Marmar [32] was administered in this study to assess perceived traumatic experiences and emotive reactions, such as intrusion, avoidance and hyperarousal, in response to the suspension of training and competitions [10]. Intrusive thoughts, as symptoms of distress, are typically associated with the re-experiencing of an event, having frequent flashbacks and difficulty in sleeping [33]. However, different types of recurrent thinking identify different conditions: repetitive thoughts associated with a stressful event are symptoms of distress, while frequently pondering or meditating on information, with more controlled thoughts focused on making sense of an experience, problem solving and anticipation, could help to overcome problems [34,35]. Avoidance reflects attempts to actively remove from the consciousness thoughts and emotions associated with a traumatic experience, such as the active defensive reaction. Hyperarousal is a high state of activation following stressful events, including symptoms such as anger, irritability, difficulty in concentration and hypervigilance.

A great number of previous studies focused on the impact of sports activities’ suspension on athletes [10]; however, little or nothing has been taken into consideration regarding disabled athletes.

The aim of this survey, carried out within a sample of disabled athletes, was to provide evidence about the stress perception and emotional reactions related to sports activities’ suspension during the first wave of the COVID-19 pandemic and to determine whether the interruption of physical activity and sport could represent a potentially traumatic event for this special population and induce post-traumatic stress disorders. It is helpful to describe the perceived impacts of quarantine among disabled athletes regarding the transactional interpretation of stress and related emotional responses in order to implement home-based interventions during lockdown and recovery protocols during the resumption of sports and recreation activities.

## 2. Materials and Methods

### 2.1. Study Design and Participants

A cognitive survey was administered during the first phase of the Italian lockdown period (12 March 2020, to 3 May 2020) using an online platform (Google Form, Google, Mountain View, CA, USA). Participants, disabled athletes and athletes were recruited with a snowball sampling strategy. The only inclusion criterion was to be affiliated with a national federation and/or a sports association. Subjects with mental disabilities were excluded from the study. A total of 146 self-selected volunteers were included in this study: 73 disabled athletes (aged 42.11 ± 13.70) and 73 athletes (aged 40.23 ± 13.73).

Disabled athletes involved in the study were those blind (52%), with mobility impairment (34%) such as limb amputation and deaf (14%).

The first section of the survey aimed to assess sociodemographic factors, such as age (emerging adults: 18–36; adults: ≥37), gender, type of sport (individual and team sports) and technical level (low, middle and high). Regarding technical level, low level included athletes who trained twice per week, middle level included athletes who trained more than twice per week and high level included athletes in national competitions organized by the national federations. Sample characteristics are shown in Table 1.

In the second section, the Impact of Event Scale-Revised (IES-R) questionnaire [25] was administered to evaluate psychological distress due to sports activities’ interruption. Participants were asked to complete the questionnaire according to emotional reactions related to sports activities’ suspension during the first lockdown period. The IES-R was validated in Italy by Craparo and colleagues [36].

A cover letter reporting the nature of the research, assurance of confidentiality and anonymity was added before the questionnaire. Electronic informed consent was obtained from all participants via email. The study was designed and conducted in accordance with the Declaration of Helsinki and approved by the bioethical local committee of the University of Rome “Foro Italico” (University Committee for Research (CAR-IRB), Code: CAR 55/2020).

### 2.2. Screening Questionnaire

The IES-R is a self-administered questionnaire that includes 22 items with a Likert rating scale from 0 (not at all) to 4 (often). The questionnaire was designed to assess current subjective distress. A total score (TS) range of greater than 32 (cutoff), out of a maximum of 88, indicates distress associated with a specific event. The questionnaire includes three subscales aimed at evaluating the symptoms of intrusion (INT) (recurring thoughts, images and feelings of the traumatic experience), avoidance (AV) (attempts to remove thoughts or emotions from consciousness) and hyperarousal (HYP) (hypervigilance, irritability and low concentration) [37]. The IES-R has a high internal consistency for INT (α: 0.87 to 0.92), AV (α: 0.84 to 0.85) and HYP (α: 0.79 to 0.90) [38]. It has also a good test–retest correlation, respectively: INT (0.57 to 0.94), AV (0.51 to 0.89), and HYP (0.59 to 0.92) [38].

### 2.3. Statistical Analysis

Data analysis was performed using SPSS statistics version 23 (IBM) software. The normal distribution of continuous variables was verified by the Kolmogorov–Smirnov test. Data are presented as mean and standard deviations. To evaluate differences among IES-R total score and IES-R subscales, as dependent variables, and age, gender, type of sport and technical level, as independent variables, a multivariate analysis of variance (MANOVA) test was used. Post-hoc comparisons were performed using the Bonferroni test. The significant level for statistical significance for all variables was set at 0.05. Cohen’s d effect size was calculated for statistically significant differences: values below 0.2 and 0.49 were considered a small effect and 0.5 and 0.79 the medium effect, ≥0.8 [39].

## 3. Results

A total of twenty-eight participants (19.18%) among the 146 respondents showed stress symptoms related to sports interruption during the COVID-19 quarantine. Of the disabled athletes and athletes, 8.22% and 30.14% had an IES-R TS higher than the cutoff, respectively (Table 2).

### 3.1. Disabled Athletes

MANOVA showed significant differences in the IES-R TS for age, where emerging adults had a higher level of stress than adults (*p* = 0.031). Significant differences were found for type of sport, where individual sports participants had a higher level of stress than team sports participants (*p* = 0.049). No significant differences were found in the IES-R TS for gender and technical level.

Regarding the IES-R subscales, significant differences were found in INT and AV for age, the emerging adults had higher scores than adults (*p* = 0.018 and *p* = 0.046, respectively). Significant differences were found in INT and HYP for type of sport, where the individual sports group had higher scores than the team sports group (*p* = 0.05 and *p* = 0.014, respectively) (Table 3).

### 3.2. Comparison between Disabled Athletes and Athletes

Comparing disabled athletes and athletes, MANOVA showed significant differences in IES-R TS and all the subscales, where athletes had higher scores than disabled athletes (*p* < 0.001 for all) (Table 4).

## 4. Discussion

This study aimed to assess the psychological condition of disabled athletes in the time of the COVID-19 pandemic and quarantine, which caused the suspension of sports activities at every level and provided data to focus attention on the emotional responses and needs of disabled athletes during this global phenomenon.

The results indicated that 8.22% of participants declared subjective distress due to the psychological impact of the COVID-19 lockdown. Previous challenges, such as difficulties related to their disability, may equip disabled people to manage the pressures of their problems, maintain a stable equilibrium [40] and become stronger and more resilient [30]. Moreover, sports engagement, in particular for disabled athletes, could provide a stress-buffering effect, inducing a sense of self-efficacy that helps athletes to cope with stressors [41]. For those with disabilities, engaging in sports can help to create habits and benefits that will last long into their life. Training efforts, competitions and the confidence of winning strengthen their positive self-perception, enhancing their self-esteem [42]. This condition could be defined as a sort of post-traumatic growth [43].

In line with other recent findings [19], individuals with disabilities reported moderate levels of stress related to the COVID-19 pandemic, probably due to the high coping strategies that they showed [44,45]. Previous studies showed that disabled athletes have a strong identity as athletes, learning to be focused on their goals [6,46]. Specifically, in our results, avoidance was found to be the common emotional response among young participants, demonstrating that younger athletes had a tendency to deny the implications of COVID-19.

Different from previous studies on gender differences in stress perception, women did not appear significantly more vulnerable to distress than men [47]. This was a somewhat surprising finding, considering that in the general population, women showed worse adaptive behaviors to potential distress for traumatic events [48]. Perhaps the experience of living with impairment developed, both in men and women, better resilience, arising from psychological strength in the process of adapting and overcoming stressful environmental demands [49].

Regarding age, emerging adult respondents experienced higher levels of self-reported stress, compared with the adult group. Young people seem to be clingier in their behaviors and daily routines [50] and are more susceptible to experience distress and social disadvantages such as discrimination and bullying and, consequently, are at risk of developing psychological concerns [51]. Moreover, younger disabled performers developed symptoms of anxiety, experiences of confusion and several concerns regarding this unknown and uncertain situation, since they do not clearly understand what is happening around them. Providing age-appropriate information about the social and health relevance of COVID-19 restrictions could reduce their anxiety [52,53]. As in a previous study [54], youngsters showed more avoidance and intrusion, as distressing symptoms, than adults. The use of avoidant strategies, with several attempts to actively remove thoughts and avoid situations that are reminiscent of a stressful situation, are common in young people [55]. The presence of high levels of intrusive thoughts in young people is likely to be associated with increased distress and an early failure to cope effectively [32,56,57]. Nevertheless, these two symptoms may represent two different phases of psychological reaction to stressful events: intrusive thoughts are generally the initial phase, followed by avoidance [58], which mitigates the negative effects of intrusion [59]. Therefore, subjects with high avoidance could be in an advanced phase of overcoming distress. Thus, coping styles focusing on the acceptance of difficult life events were thought to be crucial for psychosocial adjustment [60].

Previous findings demonstrated that avoidance may be an effective means to reduce distress in the short term [61] but, with time, may result in a decreased capacity to manage disease-related stressors in the long term [62]. As our results underlined, participants may have benefited from adopting avoidance responses as a useful mechanism to escape from experiencing negative emotions related to the COVID-19 pandemic. Taking into consideration that COVID-19 is an evolving stressor for everyone, future researchers should examine the important role of interpretational responses related to distress and whether one will be related to well-being in the short or long term, especially among individuals with disabilities.

Individual-sport disabled athletes were more stressed than team-sport disabled athletes, who showed better skills in managing anxiety and stress control than individuals. Perhaps frequent contact with teammates represents a protective factor toward the home lockdown [63]. Moreover, the present data also showed that those who are individual athletes had the highest hyperarousal and intrusion symptoms than those who are team athletes. Nevertheless, if the intrusive thoughts were the product of “deliberate rumination” on a stressful event, it could lead to better understanding the implications of such an event allowing “post-traumatic growth”. This condition could be facilitated by external support, useful for them especially in this period [64].

Comparing disabled athletes with a sample nondisabled athletes of the same age, this study showed that disabled athletes are significantly less stressed than nondisabled athletes. As a previous study suggested, disabled athletes develop a mental adaptation capacity and a positive outlook that help them to cope with their sports participation interruption. Some of them considered home confinement as an opportunity to self-explore, without the pressure from their sports training routine and competitions, to develop a stronger athletic identity [6].

Significant differences were found also in the IES-R subscales, in which nondisabled athletes showed higher results than disabled athletes for all subscales. The responses to the three subscales of IES-R indicated that the training suspension was “not at all” distressing for disabled athletes. The low emotional response and psychological impact of the “sportive stoppage” on disabled athletes is easily explainable. For people with physical disabilities, the perception of the situations that they have to face is profoundly different from the nondisabled population and may influence emotions and behaviors. Although sport represents a fundamental tool to maintain a good level of fitness and health and social contacts, it does not represent the main challenge for their life [65]. Disabled people, who were trained in their lives to achieve resilience, demonstrate persistence, a high level of motivation and determination to overcome their challenges [66]. Disability “in sè” is a challenge requiring the development of personal and social competences, and resilience gives more opportunities for rehabilitation and maintains the psychological equilibrium [67]. Therefore, athletes with disabilities had an adequate level of emotional management and consequently developed coping strategies that enabled them to cope with these temporary difficulties [68].

## 5. Conclusions

The evaluation of the impact of training suspension and compliance with restrictions on sports activities in disabled athletes confirmed the resilience of this population to adverse events. This condition is due to both the effectiveness of their engagement in athletic performance, which could act as a buffer to distress, inducing a sense of self-efficacy, self-acceptance and personal growth, and the experience of disabled people living with impairment and overcoming the social marginalization and structural barriers of a society not yet sufficiently adapted to their needs.

Deeper interactions and communications, engagement in team sports activities that provide social support, are important strategies to help disabled people in coping with the partial or complete stoppage of their sports participation and could be a good solution to alleviate emotional and psychological distress. The attempt of younger disabled athletes and individual sport athletes to understand the meaning of the loss of sports participation by recurrent thoughts may be potentially beneficial and useful in alleviating distress [69].

Home confinement could represent further marginalization for disabled athletes, considering that they could be identified as more vulnerable groups than other athletes. Therefore, lockdown-related measures can increase existing health and socioeconomic disparities if no preventive actions centered on the most vulnerable are taken as countermeasures. However, in this survey, disabled athletes demonstrated a greater ability to positively react to adversity, since sports-related activities could become a part of an individual’s cognitive–affective resources and background. Hopefully, as research on disabled people expands, it will also provide knowledge on how individuals with disabilities find ways to cope and develop positive implications from the condition of disability.

## Figures and Tables

**Table 1:** Sample characteristics.

Variable	*n* (%)
**Total**	146 (100.0)
**Role**	
Paralympics	73 (50.0)
Athletes	73 (50.0)
**Gender**	
**Paralympics**	
Male	52 (35.6)
Female	21 (14.4)
**Athletes**	
Male	52 (35.6)
Female	21 (14.4)
**Age (mean ± SD)**	41.17 ± 13.70
** Paralympics (mean ± SD)**	42.11 ± 13.70
Emerging adults (18–36)	31 (21.2)
Adults (≥37)	42 (28.8)
** Athletes (mean ± SD)**	40.23 ± 13.73
Emerging adults (18–36)	30 (20.5)
Adults (≥37)	43 (29.5)
**Sports**	
** Paralympics**	
Individual	35 (24.0)
Team	38 (26.0)
** Athletes**	
Individual	38 (26.0)
Team	35 (24.0)
**Technical Level**	
**Paralympics**	
Low level	26 (17.8)
Middle level	22 (15.1)
High level	25 (17.1)
**Athletes**	
Low level	15 (10.3)
Middle level	36 (24.6)
High level	22 (15.1)

**Table 2:** IES.R total score (IES-R TS) questionnaires (mean ± SD).

Total Score	Disabled Athletes		Athletes	
	*n* (%)	Mean ± SD	*n* (%)	Mean ± SD
Lower than the cutoff	67 (91.78%)	10.52 ± 9.06	51 (69.86%)	17.00 ± 8.22
Higher than the cutoff	6 (8.22%)	40.83 ± 7.65	22 (30.14%)	45.18 ± 12.36

**Table 3:** Comparison among disabled athletes for IES-R total score and IES-R subscales.

Variable	Groups	Mean ± SD	*p*-Value	*F*-Value
**Total score**	**Gender**			
	Male—Female	11.36 ± 10.67—17.05 ± 14.97	0.073	3.310
	**Age**			
	Emerging adults *—Adults	16.58 ± 12.40—10.38 ± 11.55	0.031 *	4.827
	**Sports**			
	Individual *—Team	15.94 ± 13.69—10.32 ± 10.16	0.049 *	4.019
	**Technical level**			
	High level—Middle level	12.24 ± 14.01—15.59 ± 12.17	0.389	0.756
	High level—Low level	12.24 ± 14.01—11.58 ± 10.46	0.849	0.037
	Low level—Middle level	11.58 ± 10.46—15.59 ± 12.17	0.225	1.511
**Intrusion**	**Gender**			
	Male—Female	4.60 ± 4.68—6.76 ± 6.01	0.106	2.681
	**Age**			
	Emerging adults *—Adults	6.87 ± 6.08—4.00 ± 4.05	0.018 *	5.859
	**Sports**			
	Individual *—Team	6.23 ± 5.42—4.29 ± 4.82	0.05 *	4.020
	**Technical level**			
	High level—Middle level	5.00 ± 5.99—6.18 ± 5.60	0.490	0.483
	High level—Low level	5.00 ± 5.99—4.62 ± 3.88	0.786	0.075
	Low level—Middle level	4.62 ± 3.88—6.18 ± 5.60	0.260	1.299
**Avoidance**	**Gender**			
	Male—Female	3.85 ± 3.86—5.71 ± 6.76	0.141	2.215
	**Age**			
	Emerging adults *—Adults	5.71 ± 4.82—3.41 ± 4.77	0.046 *	4.126
	**Sports**			
	Individual—Team	5.20 ± 5.71—3.63 ± 3.93	0.173	1.894
	**Technical level**			
	High level—Middle level	4.60 ± 5.46—5.05 ± 4.83	0.770	0.087
	High level—Low level	4.60 ± 5.46—3.62 ± 4.90	0.483	0.500
	Low level—Middle level	3.62 ± 4.90—5.05 ± 4.83	0.291	1.140
**Hyperarousal**	**Gender**			
	Male—Female	2.94 ± 3.11—4.57 ± 4.84	0.091	2.930
	**Age**			
	Emerging adults—Adults	4.00 ± 3.79—2.98 ± 3.67	0.249	1.350
	**Sports**			
	Individual *—Team	4.51 ± 4.40—2.40 ± 2.66	0.014 *	6.318
	**Technical level**			
	High level—Middle level	2.64 ± 3.82—4.36 ± 3.99	0.138	2.284
	High level—Low level	2.64 ± 3.82—3.35 ± 3.36	0.486	0.493
	Low level—Middle level	3.35 ± 3.36—4.36 ± 3.99	0.343	0.920

Emerging adults: 18–36 years; Adults: ≥37 years; *: Significant differences.

**Table 4:** Comparison between disabled athletes and athletes for IES-R TS.

Variable	Groups	Mean ± SD	*p*-Value	*F*-Value	Cohen’s d
**Total score**	Disabled athletes—Athletes *	13.01 ± 12.23—25.49 ± 16.16	<0.001 *	27.686	0.870
**Intrusion**	Disabled athletes—Athletes *	5.22 ± 5.18—10.63 ± 6.79	<0.001 *	29.348	0.896
**Avoidance**	Disabled athletes—Athletes *	4.38 ± 4.90—8.55 ± 6.15	<0.001 *	20.486	0.750
**Hyperarousal**	Disabled athletes—Athletes *	3.41 ± 3.73—6.32 ± 4.31	<0.001 *	18.948	0.722

*: Significant difference (*p* < 0.05).

## Data Availability

Data that support the findings of this study are available from the corresponding author upon reasonable request.
